# Minutes of the Second Annual Meeting of the Ohio Dental College Association, Held in February, 1853

**Published:** 1853-04

**Authors:** 


*Minutes of the second Annual Meeting of the Ohio Dental College Association, held in February,* 1853.

The Association held a preliminary meeting in the hall of the Ohio Dental College, on Friday afternoon, February 18th, 1853. On calling the roll, the following members were found to be present, viz: Dr. James Taylor, H. R. Smith, Dan. Dougherty, J. P. Ulrey, C. Bonsall, Edward Taylor, W. C. Duncan, J. Taft, Samuel Griffith, W. H. Goddard, H. E. Peebles, Jno. Allen, G. L. Van Emon, and Thomas Wood.

The minutes of last year having been first read, it was, on motion of Dr. Edward Taylor,

*Resolved,* That Drs. Taft, Peebles, and Bonsall, be appointed to fill vacancies in the examing committee, occasioned by the absence of Drs. Somerby, Hale, and Talbert.

Dr. Taft who had been appointed to read an essay at this meeting, reported his having been unable to prepare one, and was excused.

On motion of Dr. Smith, adjourned, to meet at 8½ o'clock to-morrow morning.

---

MORNING SESSION.

Met at 8½ A. M. on Saturday morning, February 19th, 1853, present, Jas. Taylor, C. Bonsall, Taft, Wood, Peebles, Smith, Duncan, Allen, Ulrey, Dougherty, and Van Emon. The minutes of yesterday were read and approved.

The committee appointed last year, to prepare By-Laws, reported having nothing to offer, and were discharged from the further consideration of the subject.

On motion of Dr. Smith, it was resolved, that hereafter the Annual meetings of the Association, be held on the 3d Monday in February of each year, at 2 o'clock, P. M. and that the exercises of the College commencement, be on the Wednesday evening following.

On motion of Dr. Taft, it was made the order of the day for 12 M. this day, to go into an election of officers for the ensuing year.

A letter having been received from Dr. Geo. Mendenhall, offering his resignation as Professor of Pathology and Therapeutics, it was on motion of Dr. Allen accepted, and the following resolution adopted.

*Resolved*, That a vote of thanks be tendered to Dr. Mendenhall, for the very efficient aid he has rendered this institution, during his connexion with the same.

On motion, of Dr. Peebles, it was *Resolved*, That this Association recommend to the Board of Trustees, Dr. J. B. Smith, as a suitable person to fill the chair of Pathology and Therapeutics, vacated by the resignation of Professor Mendenhall.

On motion of Dr. Taft, it was *Resolved*, That we recommend to the Board of Trustees, that they substitute a chair of Professor of Mechanical Dentistry, instead of appointing a Demonstrator of the same.

The subject of a change in the organization of the faculty so as to increase the duties of the demonstrator of mechanical dentistry, and change his title to that of Professor, being under discussion, a strong feeling of anxiety for the improvement of the school was shown generally by the stockholders present; and the following communication having been received from Dr. Van Emon, viz; "I would respectfully tender my resignation as Demonstrator of Mechanical Dentistry, in the Ohio College of Dental Surgery."

Signed, J. D. VAN EMON.

It was on motion of Dr. Bonsall, *Resolved*, That we now go into an election for a suitable person to recommend to the Board of Trustees to fill the chair of Professor of Mechanical Dentistry, when it appeared that Dr. H. R. Smith of Terre Haute, Ind., was selected for that purpose.

At 12 M. went into an election, when the following gentlemen were found to be elected.

Dr. James Taylor, *President*; Dr. J. Taft, *1st Vice*

*President*, Dr. Thomas Wood, 2*d Vice President;* Dr. Charles Bonsall, *Secretary;* Dr. Jno. Allen, *Treasurer;* Drs. H. E. Peebles, W. H. Goddard, A. S. Talbert, *Dental Examiners;* Geo. Mendehall, and J. S. Dodge, *Medical Examiners.*

On motion, adjourned to meet at 8½ P. M. this evening.

---

EVENING SESSION.

Met at 8½ P. M. present, Drs. Dougherty, E. Taylor, Allen, Joseph Taylor, H. R. Smith, Duncan, Van Emon, Ulrey, Peebles, James Taylor, Bonsall, Wood, and J. B. Smith.

On motion of Dr. Peebles, it was *Resolved,* That the time be extended from three years as resolved on last year to six years for the appropriation of the income from stock, &c. to the improvement of the College buildings.

Dr. James Taylor, from the committee appointed last year to report plans and cost of improving the college buildings, having reported several proposals, there was considerable discussion had on the subject, and a general feeling in favor of postponing the main improvement for another year was shown, when on motion of Dr. Edward Taylor, the committee on improvements was authorized to make such improvements as they think proper, but not to exceed the amount of money in the Treasurer's hands.

On motion of Dr. Bonsall, the Association went into an election of five persons to act as Trustees of the college property, when the following were found to be elected. Drs. Bonsall, James Taylor, Peebles, Wood, and Allen.

Dr. J. B. Smith, of Cincinnati, Drs. J. F. Johnston, of Indianapolis, Ia., J. L. Milton, Lexington, Mo., and E. H. Eames, of Ann Arbor, Michigan, Alumni of the institution were elected members of the Association, and signed the constitution.

On motion of Dr. Wood, the Secretary was directed to hand a copy of the minutes to the editor of the "Dental Register of the West," for publication in that journal.

The minutes of the preceeding meetings were now read and approved, and on motion the Association adjourned *sine die.* CHARLES BONSALL, *Secretary.*

